# Supplementary material for: Getting More Out of Biomedical Documents with GATE's Full Lifecycle Open Source Text Analytics
Source: PLoS Comput Biol. 2013 Feb 7;9(2):e1002854. doi: 10.1371/journal.pcbi.1002854 (PMC3567135; doi:10.1371/journal.pcbi.1002854)
Supplement: Dataset S1 — GATE software. Dataset S1 bundled with this paper contains a distribution of GATE (or see http://gate.ac.uk/download/). (TGZ) [file pcbi.1002854.s001.tgz › plos-gate/plugins/Tagger_MetaMap/doc/gate/metamap/class-use/OutputMode.html]

Uses of Class gate.metamap.OutputMode


---


|  |  |  |
| --- | --- | --- |
| **Overview** \| **Package** \| **Class** \| **Use** \| **Tree** \| **Deprecated** \| **Index** \| **Help** | |  |
| PREV   NEXT | **FRAMES**    **NO FRAMES**     **All Classes** |  |


---


## **Uses of Class gate.metamap.OutputMode**

| Packages that use OutputMode | |
| --- | --- |
| **gate.metamap** |  |

Uses of OutputMode in gate.metamap

| Methods in gate.metamap that return OutputMode | |
| --- | --- |
| `OutputMode` | `MetaMapPR.getOutputMode()` |
| `static OutputMode` | `OutputMode.valueOf(java.lang.String name)`             Returns the enum constant of this type with the specified name. |
| `static OutputMode[]` | `OutputMode.values()`             Returns an array containing the constants of this enum type, in the order they are declared. |

| Methods in gate.metamap with parameters of type OutputMode | |
| --- | --- |
| `void` | `MetaMapPR.setOutputMode(OutputMode outputMode)` |

---


|  |  |  |
| --- | --- | --- |
| **Overview** \| **Package** \| **Class** \| **Use** \| **Tree** \| **Deprecated** \| **Index** \| **Help** | |  |
| PREV   NEXT | **FRAMES**    **NO FRAMES**     **All Classes** |  |


---
